# Genome-wide identification and characterization of the *NF-Y* gene family in grape (*vitis vinifera* L.)

**DOI:** 10.1186/s12864-016-2989-3

**Published:** 2016-08-11

**Authors:** Chong Ren, Zhan Zhang, Yi Wang, Shaohua Li, Zhenchang Liang

**Affiliations:** 1 Beijing Key Laboratory of Grape Science and Enology and Key Laboratory of Plant Resource, Institute of Botany, the Chinese Academy of Sciences, Beijing, 100093 People’s Republic of China; 2 University of Chinese Academy of Sciences, Beijing, 100049 People’s Republic of China

**Keywords:** Grape (*Vitis vinifera* L.), NF-Y transcription factor, Phylogenetic analysis, Expression profiles, Quantitative real-time PCR

## Abstract

**Background:**

Nuclear factor Y (NF-Y) transcription factor is composed of three distinct subunits: NF-YA, NF-YB and NF-YC. Many members of NF-Y family have been reported to be key regulators in plant development, phytohormone signaling and drought tolerance. However, the function of the NF-Y family is less known in grape (*Vitis vinifera* L.).

**Results:**

A total of 34 grape *NF-Y* genes that distributed unevenly on grape (*V. vinifera*) chromosomes were identified in this study. Phylogenetic analysis was performed to predict functional similarities between *Arabidopsis thaliana* and grape *NF-Y* genes. Comparison of the structures of grape *NF-Y* genes (*VvNF-Ys*) revealed their functional conservation and alteration. Furthermore, we investigated the expression profiles of *VvNF-Ys* in response to various stresses, phytohormone treatments, and in leaves and grape berries with various sugar contents at different developmental stages. The relationship between *VvNF-Y* transcript levels and sugar content was examined to select candidates for exogenous sugar treatments. Quantitative real-time PCR (qPCR) indicated that many *VvNF-Ys* responded to different sugar stimuli with variations in transcript abundance. qPCR and publicly available microarray data suggest that *VvNF-Ys* exhibit distinct expression patterns in different grape organs and developmental stages, and a number of *VvNF-Ys* may participate in responses to multiple abiotic and biotic stresses, phytohormone treatments and sugar accumulation or metabolism.

**Conclusions:**

In this study, we characterized 34 *VvNF-Ys* based on their distributions on chromosomes, gene structures, phylogenetic relationship with *Arabidopsis NF-Y* genes, and their expression patterns. The potential roles of *VvNF-Ys* in sugar accumulation or metabolism were also investigated. Altogether, the data provide significant insights on *VvNF-Ys*, and lay foundations for further functional studies of *NF*-*Y* genes in grape.

**Electronic supplementary material:**

The online version of this article (doi:10.1186/s12864-016-2989-3) contains supplementary material, which is available to authorized users.

## Background

NF-Y (for Nuclear factor Y) transcription factors (TFs) are almost found in all eukaryotes, and they are involved in regulation of gene expression by binding the CCAAT element [1, 2]. The NF-Y complex known as CCAAT binding factor (CBF) or heme activator protein (HAP) consists of three distinct subunits: NF-YA (also known as CBF-B or HAP2), NF-YB (CBF-A or HAP3) and NF-YC (CBF-C or HAP5) [3]. All subunits contain evolutionarily-conserved DNA binding and subunit interaction domains to form heterotrimeric complexes [4–6]. Notably, the NF-YB proteins without a nuclear localization signal (NLS) have to interact with NF-YC in the cytoplasm to translocate into the nucleus, where the heterodimer is combined with NF-YA to form the final heterotrimer [7, 8]. Despite the ubiquity of NF-Y proteins in eukaryotes, there is only one or two genes encoding each NF-Y subunit in animals and yeast [9, 10]. In contrast, there are multiple genes encoding each subunit in plants [10, 11]. For example, 10 NF-YAs, 13 NF-YBs, and 13 NF-YCs are encoded by the *Arabidopsis thaliana* genome [1, 10]. This expansion is a common feature in the plant kingdom, and it helps plants form flexible, versatile TF systems to accommodate complex and diverse environment conditions [11].

As a kind of combinatorial TFs, NF-Ys have been reported to be involved in regulation of plant development and respond to various abiotic and biotic stresses [12–19]. The *Arabidopsis LEAFY COTYLEDON 1* (*LEC1*, *AtNF-YB9*) is the first cloned and well-known plant *NF*-*Y* gene, and it has been proven that LEC1 is a pivotal regulator in embryogenesis [12, 13, 20, 21]. Recently, the *NF-Y* genes are also found to be involved in response to endoplasmic reticulum (ER) stress [22, 23]. Grape (*Vitis* spp.) is cultivated worldwide and has tremendous economic value, and a few reports have emerged revealing the role of *VvL1L* in grape [24, 25]. However, the function of the overwhelming majority of *NF-Y* genes in grape is still poorly understood, despite the conservation of functional amino acid residues across different species [26–28].

To explore and characterize the potential functions of grape *NF-Y* genes (*VvNF-Ys*), we adopted bioinformatics to analyze the 34 identified *VvNF-Ys* (8 *NF-YAs*, 18 *NF-YBs*, 8 *NF-YCs*) based on publicly available data. Furthermore, we investigated the expression patterns of *VvNF-Ys* in response to different biotic and abiotic stresses, exogenous phytohormone, and sugar treatments. In addition, the expression profiles of *VvNF-Ys* in grape berries were examined at different developmental stages. The phylogenetic analysis of *NF-Ys* from grape and *Arabidopsis*, investigation of protein motif and exon-intron structure patterns, and the experimental data provide insights on the function of *VvNF-Ys*. Taken together, our results provide a set of candidate *NF-Y* genes for future study and genetic modification in grape.

## Results

### Identification and characterization of grape *NF-Y* genes

NF-Y proteins were identified by searching the Plant Transcription Factor Database (PlantTFDB, http://planttfdb.cbi.pku.edu.cn/) and the UniProt database (http://www.uniprot.org/) using the PFAM and KOG IDs of conserved domains. Then, a BLAST search of the 12× grape genome was performed using full-length amino acid sequences of candidate *NF-Y* genes. By removing incomplete and redundant sequences, 34 *NF-Y* genes were identified, including 8 *NF-YA*, 18 *NF-YB*, and 8 *NF-YC* genes (Table 1). The 34 *VvNF-Ys* were named based on their distribution and relative distance on grape chromosomes. Thirty-two *VvNF-Ys* could be mapped on 14 grape chromosomes with the exception of *VvNF-YB17* and *VvNF-YB18* (Table 1 and Additional file 1: Figure S1). Among these chromosomes, four possessed only one *NF-Y* gene, and seven possessed two *NF-Y* genes. Chromosomes 6 and 19 had five *NF-Y* genes, most of which were concentrated in the upper part of the chromosomes (Additional file 1: Figure S1). Uneven and variable distribution of *VvNF-Ys* on grape chromosomes is consistent with the results of previous reports [3, 29].

**Table 1:** NF-Y transcription factors in grape

Name	Gene ID	Best match in Arabidopsis	Chr.	Strand	Genomic (bp)	No. of aa	pI
NF-YA Subunit							
NF-YA1	GSVIVT01036936001	At1g31420	2	+	51220	1611	8.89
NF-YA2	GSVIVT01025252001	At5g12840, AtNF-YA1	6	+	7100	306	6.49
NF-YA3	GSVIVT01033313001	At3g20910, AtNF-YA9	8	+	5639	354	8.67
NF-YA4	GSVIVT01022601001	At5g06510, AtNF-YA10	8	_	8762	309	9.34
NF-YA5	GSVIVT01016790001	At1g72830, AtNF-YA3	9	+	7541	336	9.15
NF-YA6	GSVIVT01021622001	At1g30500, AtNF-YA7	10	+	14679	208	7.17
NF-YA7	GSVIVT01015120001	At3g14020, AtNF-YA6	11	+	2972	310	8.76
NF-YA8	GSVIVT01032101001	At3g20910, AtNF-YA9	13	_	8954	405	9.30
NF-YB Subunit							
NF-YB1	GSVIVT01010260001	At1g09030, AtNF-YB4	1	+	459	152	5.78
NF-YB2	GSVIVT01010264001	At1g09030, AtNF-YB4	1	+	459	152	8.87
NF-YB3	GSVIVT01017741001	At2g47810, AtNF-YB5	5	_	417	138	5.16
NF-YB4	GSVIVT01036120001	At2g27470, AtNF-YB11	6	+	8280	193	5.59
NF-YB5	GSVIVT01025110001	At3g53340, AtNF-NB10	6	_	2009	133	6.83
NF-YB6	GSVIVT01022214001	At5g64950	7	+	876	291	9.07
NF-YB7	GSVIVT01010959001	At2g47810, AtNF-YB5	7	+	387	128	6.84
NF-YB8	GSVIVT01025539001	At2g37060, AtNF-YB8	8	+	2436	161	5.81
NF-YB9	GSVIVT01030085001	At4g12730	12	+	7125	482	5.93
NF-YB10	GSVIVT01016347001	At2g37060, AtNF-YB8	13	+	4682	176	6.42
NF-YB11	GSVIVT01031089001	At1g09030, AtNF-YB4	14	+	444	147	6.83
NF-YB12	GSVIVT01008215001	At5g23090, AtNF-YB13	17	_	8526	155	4.62
NF-YB13	GSVIVT01014672001	At5g55660	19	+	11644	1098	6.36
NF-YB14	GSVIVT01014673001	At5g47640, AtNF-YB2	19	+	1711	210	6.44
NF-YB15	GSVIVT01014689001	At5g47670, AtNF-YB6	19	+	1402	209	5.89
NF-YB16	GSVIVT01014690001	At5g47670, AtNF-YB6	19	+	1537	215	5.48
NF-YB17	GSVIVT01004375001	At4g14540, AtNF-YB3	Un	+	1376	114	4.10
NF-YB18	GSVIVT01002895001	At5g47670, AtNF-YB6	Un	_	2458	211	5.91
NF-YC Subunit							
NF-YC1	GSVIVT01019784001	At1g54830, AtNF-YC3	2	+	351	116	9.46
NF-YC2	GSVIVT01017901001	At1g08970, AtNF-YC9	5	+	4358	104	9.10
NF-YC3	GSVIVT01025169001	At1g07980, AtNF-YC10	6	+	8437	425	9.72
NF-YC4	GSVIVT01037394001	At3g12480, AtNF-YC11	6	_	8197	301	4.92
NF-YC5	GSVIVT01036581001	At3g12480, AtNF-YC11	13	+	15144	271	9.40
NF-YC6	GSVIVT01030963001	At5g63470, AtNF-YC4	14	_	946	129	6.36
NF-YC7	GSVIVT01008570001	At3g48590, AtNF-YC1	17	_	4379	215	4.95
NF-YC8	GSVIVT01036760001	At1g56170, AtNF-YC2	19	_	1072	114	5.06

Characteristics of the 34 *VvNF-Ys* are shown in Table 1. Significant difference of the length of *VvNF*-*Y* sequences was observed, with a range from 351 to 51,220 bp, and the difference results in variability of predicted amino acid numbers. The exon-intron structures of *VvNF*-*Ys* were also analyzed (Additional file2: Figure S2). The exon-intron organization could indicate the evolutionary relationships within multi-gene families [30]. Most of *VvNF-YAs* had five or six exons and four or five introns and their intron phases occurred in the same pattern except for *VvNF-YA1* and *VvNF-YA8*. The structures of *VvNF-YBs* and *VvNF-YCs* were more variable and complicated, and the two families shared similar exon-intron organization (Additional file 2: Figure S2). The results were consistent with the previous report [29].

### Phylogenetic analysis and multiple alignment of NF-Y protein sequences

**Fig. 1:**
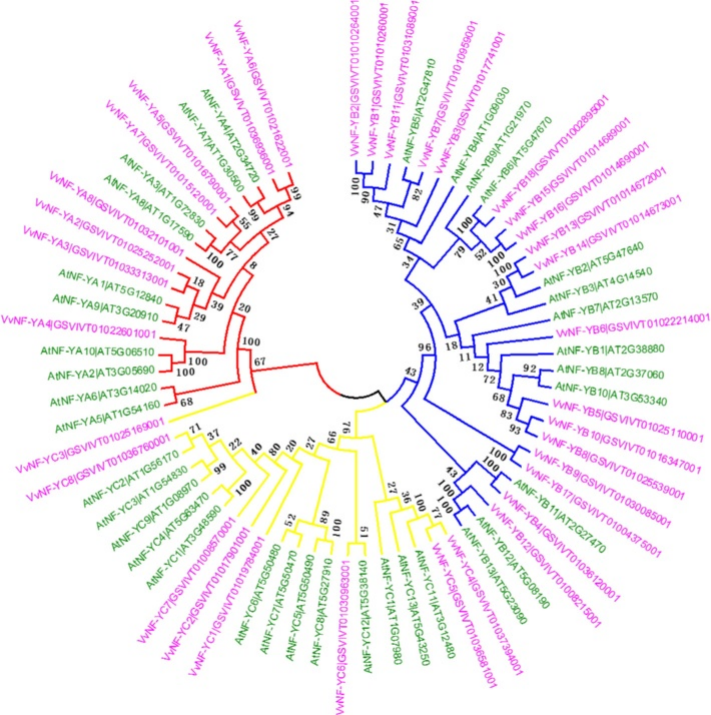
Phylogenetic analysis of NF-Y proteins from grape and *Arabidopsis thaliana*. Thirty-four NF-Y proteins from grape and 36 NF-Y proteins from *Arabidopsis* were divided into four branches according to subunit type. Red branch indicates NF-YAs, blue branch represents NF-YBs, and two yellow branches denote NF-YCs

To investigate the evolutionary relationship and functional association of *VvNF-Ys* with *Arabidopsis NF-Y* family, we constructed an unrooted phylogenetic tree using the protein sequences of NF*-*Ys from grape and *Arabidopsis* (Fig. 1). The phylogenetic analysis showed that the 34 *VvNF-Ys* were divided into three groups (Fig. 1). Almost all the members of *VvNF-YAs*, *VvNF-YBs* or *VvNF-YCs* were clustered into the same sub-branch except for *VvNF-YC3*. Three pairs of *NF-YAs*, four pairs of *NF-YBs* and one pair of *NF-YCs* showed high similarity in sequence, respectively (Fig. 1). Most of *VvNF-Ys* had homologs in *Arabidopsis*. The phylogenetic relationship indicated basal architecture conservation and possible functional similarities of NF-Y family between grape and *Arabidopsis*.

**Fig. 2:**
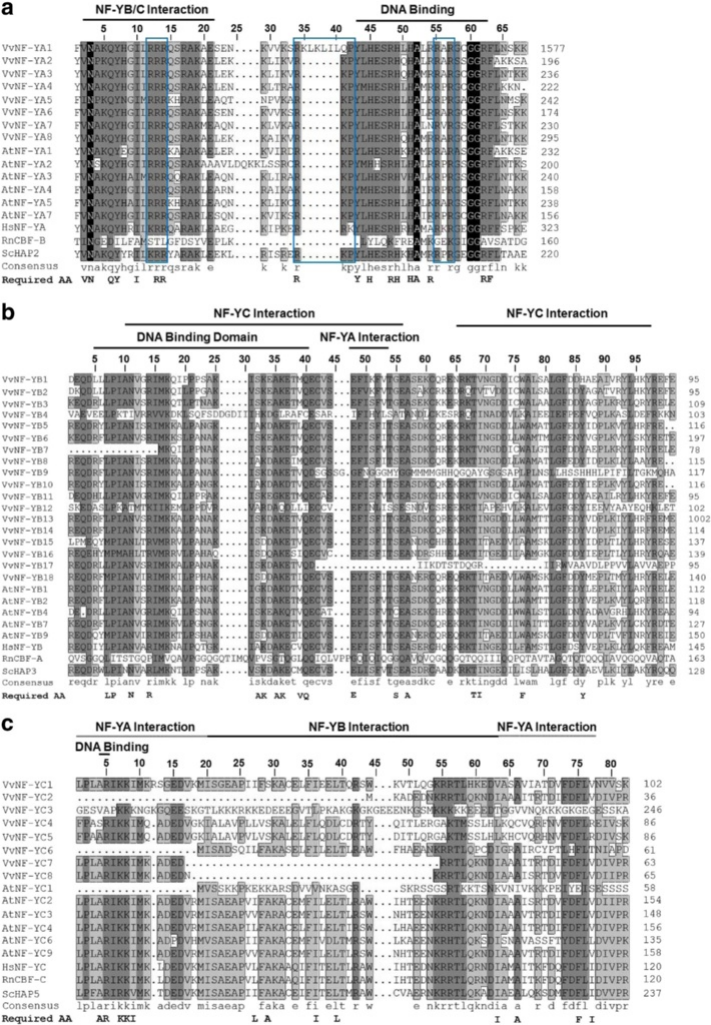
Alignments of grape NF-Y domains. The sequences of grape NF-YAs (**a**) NF-YBs (**b**) and NF-YCs (**c**) were aligned with corresponding referred sequences from *Arabidopsis thaliana* (At), human (Hs), mouse (Rn) and yeast (Sc), respectively. The actual amino acid numbers of the end of the domains are shown on the right of the figure. Functionality required amino acids (Required AA) that are verified in rat [44] and yeast [45] are given below the sequences. The residual clusters located in the blue boxes are essential for nuclear targeting [64]

Multiple sequence alignments of NF-Y proteins from grape, *Arabidopsis*, human (*Homo sapiens*), mouse (*Rattus norvegicus*) and yeast (*Saccharomyces cerevisiae*) were generated. NF-YA proteins which lack distinct homology to other annotated proteins [30] were characterized by two conserved domains: the DNA-binding domain and the subunit interaction domain [31, 32]. The two domains were conserved among plants and other eukaryote organisms (Fig. 2). The amino acid residues required for functionality in most mammals and yeast [31, 32] were present in grape NF-YA proteins (Fig. 2a). The conservation of functionality required amino acid residues across different eukaryote lineages strongly suggests functional conservation [1]. As with NF-YA proteins, NF-YBs and NF-YCs contained DNA-binding and subunit interaction domains as well (Fig. 2b, c). The required amino acids were well-conserved in most of grape NF-YB proteins except for NF-YB7 and NF-YB17. The aspartate (D_72_) which is thought to be significant for protein interactions [28, 33] was conserved in almost all NF-YB proteins (Fig. 2b). Half of grape NF-YC proteins exhibited residue deletions, and some residues in NF-YC were replaced by alternative ones of similar properties. However, the arginine (R_52_) and aspartate (D_59_) which are necessary for stabilization of NF-YB/C [28] were present in most NF-YCs (Fig. 2c). The conservation of protein sequences suggests the conserved function while the non-conservative changes would indicate novel functional alterations [1].

### Expression patterns of *VvNF-Ys* in response to abiotic, biotic stresses and phytohormones

Numerous reports have revealed the function of individual *NF-Y* genes in responses to various biotic and abiotic stresses [14, 18, 19, 34, 35]. To further investigate how *NF-Y* genes response to stresses, expression of *VvNF-Y* genes was measured under several stresses. Probe sets from Affymetrix GeneChip platform for 16 *VvNF-Ys* (Additional file 3: Table S1) were successfully obtained and corresponding genes were selected for further study.

**Fig. 3:**
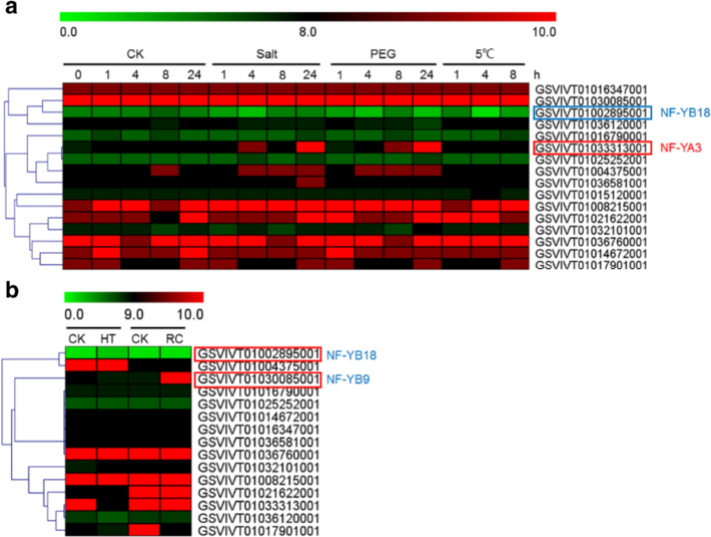
Expression profiles of *VvNF-Ys* in response to abiotic stress treatments. **a** Expression patterns of *VvNF-Ys* in response to salt, drought (PEG) and cold (5 °C) treatments. **b** Expression patterns of *VvNF-Ys* under heat stress treatment. HT represents high temperature and RC means recovery process. The color scale indicates fold-change values (log2 values) with red representing increased transcript abundance and green indicating decreased transcript abundance. A red box indicates up-regulation and blue box indicates down-regulation

We first examined the responsiveness of *VvNF-Ys* to multiple abiotic stresses including salt, drought, cold and high temperature by taking advantage of publicly available data. ‘Cabernet Sauvignon’ (*Vitis vinifera* L.) plants were treated with salt, drought (PEG) and cold (5 °C), respectively, and expression of *VvNF-Ys* was analyzed subsequently. About three *VvNF-Ys* (1 *VvNF-YA* and 2 *VvNF-YBs*) were up- or down-regulated (≥1-fold) to at least one stress treatment (Fig. 3). *VvNF-YA3* was induced and the transcript level reached a peak of nearly 2-fold at 24 h after salt and PEG treatments, while *VvNF-YB18* responded to all the stress treatments and its expression was rapidly suppressed (>2-fold) at 4 h after the treatments (Fig. 3a). The difference in expression patterns of *VvNF-YA3* and *VvNF-YB18* suggests their different roles in response to salt and PEG. For heat treatment, ‘Cabernet Sauvignon’ seedlings derived from stem cuttings were placed at 45 °C and then recovered at 25 °C [36]. Two *VvNF-YBs* responded (≥2-fold) to heat stress or the following recovery process (Fig. 3b). The transcript abundance of *VvNF-YB18* was increased (≥2-fold) in response to heat treatment, whereas the other gene, *VvNF-YB9*, was down-regulated (≥1-fold) after the heat treatment. However, the transcript level of *VvNF-YB9* was increased (≥2-fold) again during the subsequent recovery process (Fig. 3b). These results showed that *VvNF-YB18* may help enhance the resistance of grape to heat stress, and *VvNF-YB9* may participate in the heat recovery process. All the results discussed above revealed that some of *VvNF-Ys* may be associated with the signaling of abiotic stress responsiveness. Among these genes, *VvNF-YB18* responded to various stresses and showed different expression patterns upon these treatments, suggesting its different roles in multiple signaling pathways.

**Fig. 4:**
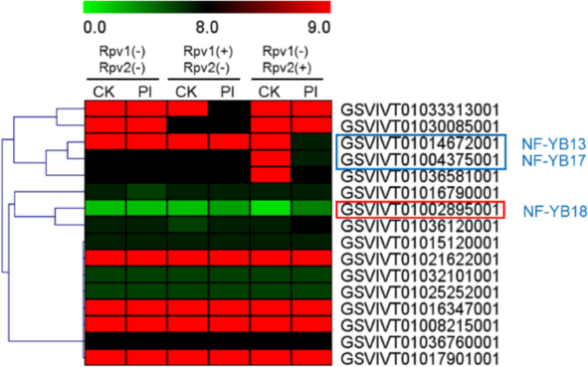
Expression patterns of *VvNF-Ys* during downy mildew infection. Plants of different genotypes were used in the experiment: susceptible plants without resistance loci *Rpv1* and *Rpv2* (Rpv1 (−) Rpv2 (−)) and incompatible plants bearing the resistance gene *Rpv1* (Rpv1 (+) Rpv2 (−)) or *Rpv2* (Rpv1 (−) Rpv2 (+)). PI means *Plasmopara viticola* inoculation. The color scale indicates fold-change values (log2 values) with red representing increased transcript abundance and green indicating decreased transcript abundance. A red box indicates up-regulation and blue box indicates down-regulation

We next analyzed the possible involvement of *NF-Y* genes in response to biotic stresses. No obvious changes (≥1-fold) in expression levels of *VvNF-Ys* were detected in ‘Cabernet Sauvignon’ after powdery mildew fungus (PM) infection (data not shown), and this result is consistent with a previous report [37]. The expression patterns of *VvNF-Ys* after *Plasmopara viticola* inoculation were also analyzed. Three *VvNF-YBs* were found to respond (≥2-fold) to *P. viticola* infection (Fig. 4). *VvNF-YB13* and *VvNF-YB17* were down-regulated in incompatible plant bearing the resistant gene *Rpv2* after *P. viticola* infection as compared to the mock control. The expression level of *VvNF-YB18* in resistant plants (bearing *Rpv1* or *Rpv2*) was increased after inoculation (Fig. 4). These results indicated that a certain number of *VvNF-Ys* displayed pathogen-related expression patterns, implying their possible involvement in grape immune signaling. Those *VvNF-Ys* with altered expression levels might be candidates for further study of grape immune response.

**Fig. 5:**
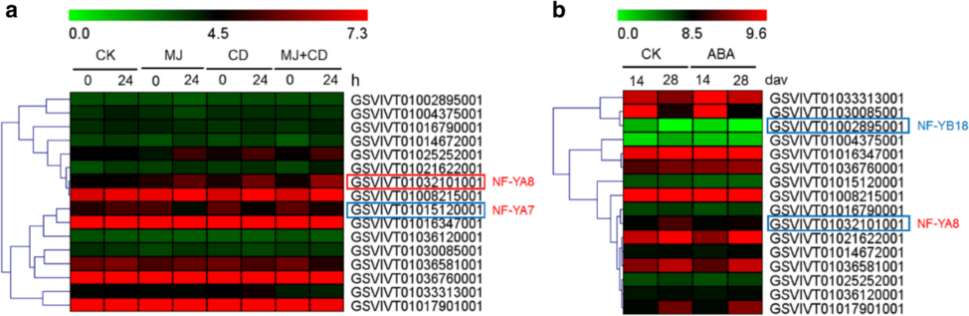
Expression patterns of *VvNF-Ys* in response to phytohormone and elicitor treatment. **a** Expression patterns of *VvNF-Ys* in response to methyl jasmonate (MJ) and cyclodextrin elicitor (CD). **b** Expression patterns of *VvNF-Ys* under ABA treatment. Dav represents days after veraison. The color scale indicates fold-change values (log2 values) with red representing increased transcript abundance and green indicating decreased transcript abundance. A red box indicates up-regulation and blue box indicates down-regulation

Phytohormones such as abscisic acid (ABA), methyl jasmonate (MJ), salicylic acid (SA), and ethylene have been reported to act as messengers in plant response to abiotic and biotic stresses [38]. To examine the influence of phytohormones on *VvNF-Ys* expression, we analyzed the transcript levels of *VvNF-Ys* in ‘Monastrell’ (*V. vinifera*) calli in response to exogenously applied MJ and cyclodextrin elicitor (CD). As shown in Fig. 5a, *VvNF-YA7* showed a decreased transcript level (≥1-fold) whereas *VvNF-YA8* showed an increased transcript level (≥1-fold) in response to exogenous MJ and CD. For ABA treatment, grapevines of ‘Cabernet Sauvignon’ at veraison were treated with 400 mg/L ABA solution. Two *VvNF-Ys* (1 *VvNF-YA* and 1 *VvNF-YB*) showed a decrease in transcript abundance upon exogenous ABA treatment (Fig. 5b). In contrast to treatments with MJ and CD, *VvNF-YA8* was slightly down-regulated (0.5-fold) upon ABA treatment at 28 day after veraison (dav). The expression of *VvNF-YB18*, however, was repressed (>1-fold) by exogenous ABA at 14 dav. The possible function of *VvNF-YB18* in ABA signaling pathway may partly account for its involvement in response to abiotic and biotic stresses.

### Expression profiles of *VvNF-Ys* in grape leaves and berries at different development stages

**Fig. 6:**
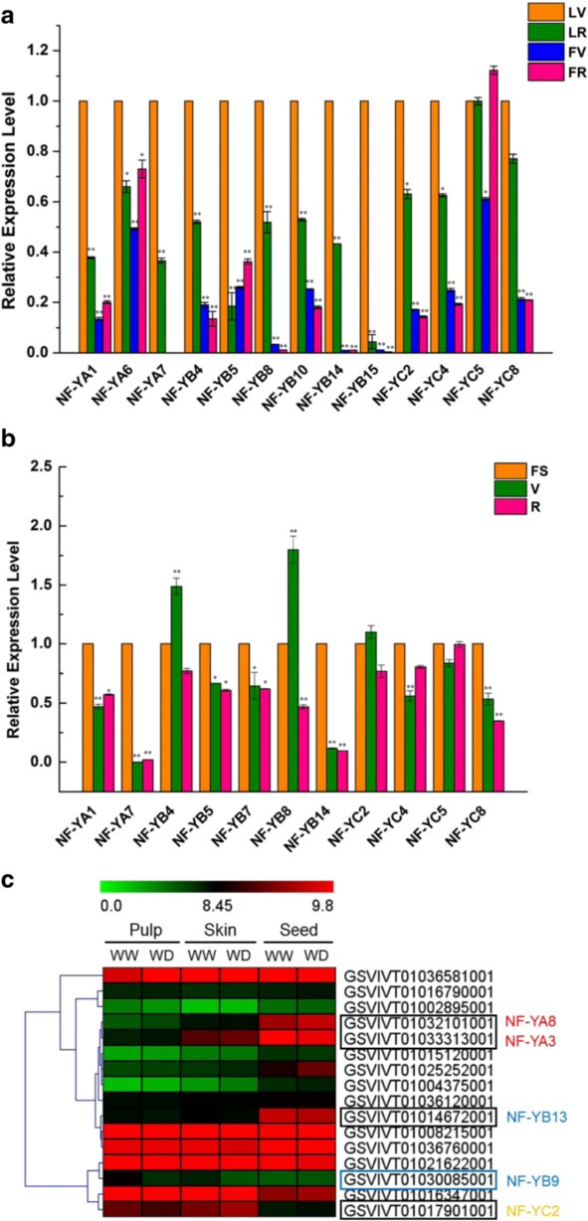
Expression profiles of *VvNF-Ys* in grape leaves and berries at different developmental stages. **a** Detailed expression levels of *VvNF-Ys* in grape leaves and berries. LV and LR denote leaves at veraison (V) and fully ripe (R) while FV and FR represent berries at V and R, respectively. **b** Detailed expression levels of *VvNF-Ys* in grape berries at three different developmental stages: fruit set (FS), V and R. **c** Expression patterns of *VvNF-Ys* in different berry tissues. WW and WD mean well-watered and water-deficit conditions, respectively. Grape *Actin1*and *UBC* were used as internal controls of quantitative real-time PCR analysis. The data are presented as mean values ± SD. * and ** represent statistically significant (*P* < 0.05) and highly significant (*P* < 0.01) differences, respectively. Significance of values in (**a**) was based on comparison of expression levels in leaves and berries at different stages with expression levels in leaves at V while in (**b**) was based on comparison of expression levels in berries at V and R with expression levels in berries at FS

To investigate expression patterns of *VvNF-Ys* in two of the most important grape organs, leaves and berries, quantitative real-time PCR (qPCR) was conducted to analyze *VvNF-Y* expression in leaves (L) and berries (F) of ‘Semillon’ (*V. vinifera*) at veraison (V) and fully ripe stage (R) (namely, LV, LR, FV and FR), respectively. However, due to the homogeneous properties of *VvNF-Ys* as well as the fact that primers did not work as well as expected, about half of the *VvNF-Ys* were selected for qPCR analyses, and 13 yielded significant results (Fig. 6a). Generally, the expression levels of *VvNF-Ys* in leaves were higher (*P* < 0.01) than that in berries, and most of *VvNF-Ys* exhibited higher transcript levels (*P* < 0.05) in LV than in LR (Fig. 6a). For example, *VvNF-YB8* was differentially expressed (*P* < 0.01) in leaves, and the transcript level of *VvNF-YB8* was much higher (*P* < 0.01) in LV as compared to that in LR (Fig. 6a). This result indicated that *VvNF-YB8* might be a tissue-specific gene and participate in synthesis-oriented biological processes. It is notable that the transcript abundance of most *VvNF-Ys* exhibited no much difference in berries with the exception being *VvNF-YC5*, which had a higher expression level (*P* < 0.05) in FR rather than in FV (Fig. 6a). All these results suggested that some of *VvNF-Ys* may be associated with grape development, which is consistent with the results of RNA-seq (data not shown). qPCR was conducted to further demonstrate the expression patterns of *VvNF-Ys* in grape berries at three different developmental stages. More than half of tested *VvNF-Ys* were predominantly expressed (*P* < 0.01) in specific development period as expected (Fig. 6b). *VvNF-YA7* and *VvNF-YB14* were differentially expressed at fruit set (FS), while *VvNF-YB4* and *VvNF-YB8* were dominantly expressed at veraison (V). The transcript level of *VvNF-YB8* was decreased (*P* < 0.01) at the development stage of fully ripe (R). These results indicated that *VvNF-Ys* might paly roles throughout the development of grape, and expression of specific genes would be regulated at certain stages of grape development.

Additionally, expression profiles of *VvNF-Ys* in different berry tissues were also detected [39]. Five *VvNF-Ys* exhibited different expression patterns in different berry tissues (Fig. 6c). Two *VvNF-YAs* (*VvNF-YA3* and *VvNFF-YA8*) and *VvNF-YB13* were differentially expressed (>2-fold) in seed, and *VvNF-YB9* as well as *VvNF-YC2* was predominantly expressed (>1.5-fold) in pulp and skin (Fig. 6c). Notably, the expression of *VvNF-YB9* in pulp and skin was affected (>1.5-fold) by water supply, suggesting its possible role in response to water deficiency.

### Expression of *VvNF-Ys* in response to different sugar content and exogenous sugar treatment

**Fig. 7:**
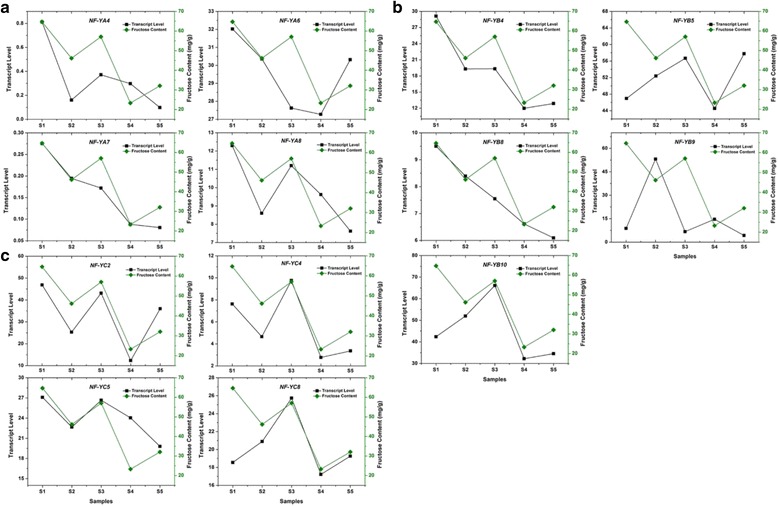
Expression levels of *VvNF-Ys* and fructose content in different grape varieties. *VvNF-YAs* (**a**) *VvNF-YBs* (**b**) and *VvNF-YCs* (**c**) are divided into three groups. Soluble sugars were extracted from five grape varieties (S1-S5) and then analyzed by HPLC with water as eluent. The fructose content was relatively stable in three successive years (unpublished data), so values for 1 year are given as reference. Black broken lines denote transcript levels of *NF-Y* genes, and green broken lines indicate fructose content in grape berries

The expression levels of some *VvNF-Ys* were increased in grape berries at veraison, which is characterized by the accumulation of hexose sugar in flesh and skin [40]. To examine whether there exists a relationship between expression of *VvNF-Ys* and sugar content, we first investigated the expression levels of *VvNF-Ys* in five grape varieties with different sugar contents (Fig. 7). The content of glucose, sucrose and fructose was measured, respectively, and sucrose was omitted from the analysis because of its extremely low content. Besides, the content of glucose was close to that of fructose (data not shown), so fructose and total sugar were finally chosen to evaluate the correlation between sugar content and *VvNF-Ys* expression. There were four *VvNF-Ys* (1 *VvNF-YA*, 1 *VvNF-YB* and 2 *VvNF-YCs*) changing their expression with the contents of fructose in all varieties. Interestingly, variation of expression levels of most *VvNF-Ys* was consistent with that of fructose contents in at least four grape varieties (Fig. 7). However, this sugar-related expression pattern of *VvNF-Ys* no longer exist if examined with total sugar contents (Additional file 4: Figure S3). These results suggested that a number of *VvNF-Ys*, such as *VvNF-YA7*, *VvNF-YB4* and *VvNF-YC2*, may tend to be responsive to specific sugars.

**Fig. 8:**
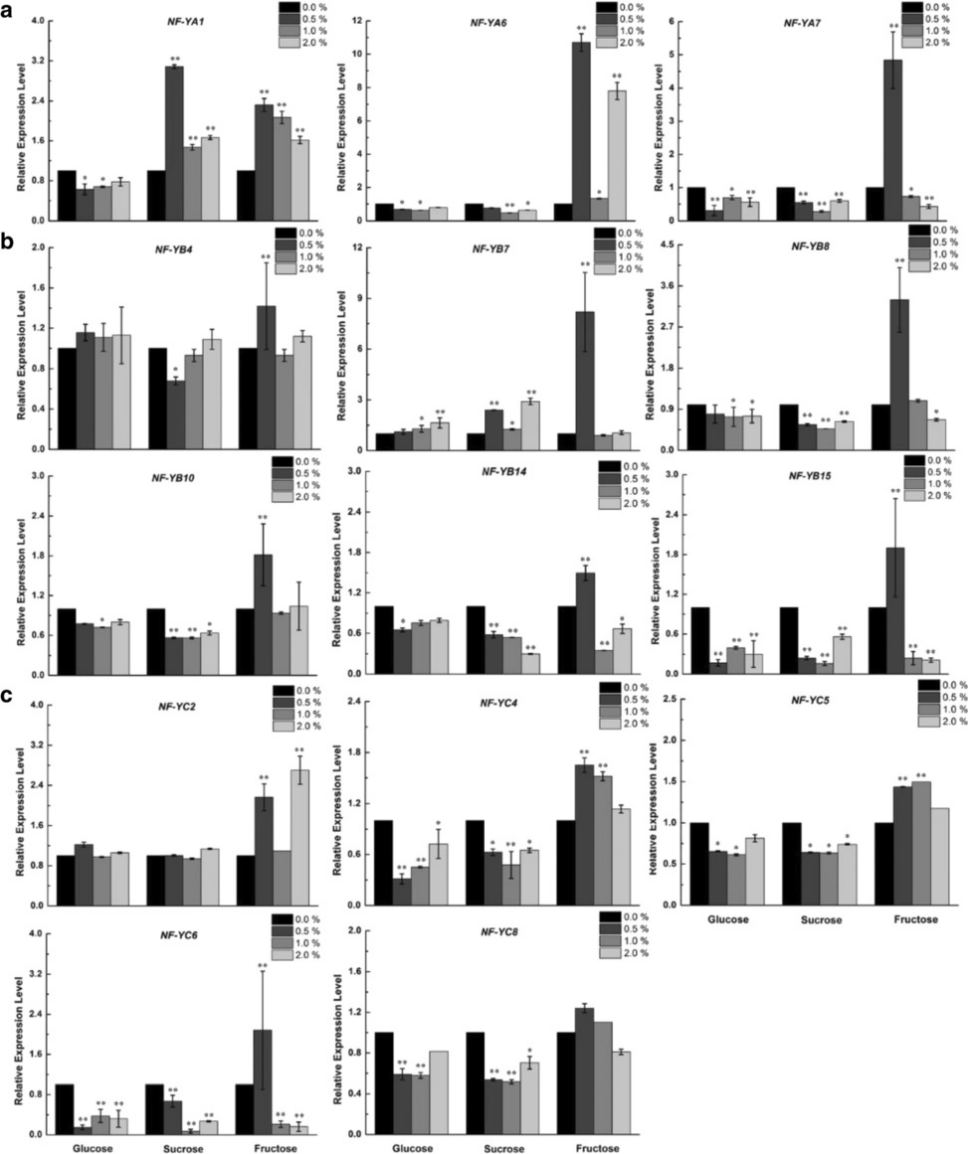
Detailed expression levels of *VvNF-Ys* in response to exogenous sugar treatments. *VvNF-YAs* (**a**) *VvNF-YBs* (**b**) and *VvNF-YCs* (**c**) are divided into three groups. Relative expression levels were measured by quantitative real-time PCR, and grape *Actin1* and *UBC* were used as internal controls. The data were showed as mean values ± SD. * and ** represent significant (*P* < 0.05) and highly significant (*P* < 0.01) differences, respectively. Significance of values was based on comparison of expression levels at 0.5, 1.0, or 2.0 % (v/v) sugar with expression levels at 0.0 % (v/v) sugar in ‘Chardonnay’ suspension cells. The experiment was repeated three times

To verify the hypothesis that some *VvNF-Ys* could respond to specific sugar, we analyzed the expression patterns of *VvNF-Ys* in ‘Chardonnay’ (*V. vinifera*) suspension cells after treatment with exogenous glucose, sucrose and fructose at a final concentration of 0.0 (CK), 0.5, 1.0, or 2.0 % (w/v), respectively. In general, all the tested *VvNF-Ys* could be induced (*P* < 0.01) by exogenous fructose except for *VvNF-YC8* (Fig. 8), and most *VvNF-Ys* were down-regulated (*P* < 0.05) in response to glucose and sucrose treatments (Fig. 8). The expression of *VvNF-YA1* and *VvNF-YB7*, however, was induced (*P* < 0.01) by sucrose treatment (Fig. 8a–b). Additionally, the expression of *VvNF-YB7* was also induced by glucose (Fig. 8b). Intriguingly, 11 out of 14 *VvNF-Ys* responded strongly to the fructose treatment at the final concentration of 0.5 % with their transcript levels increasing from 1.3-fold (*VvNF-YC8*) to more than 10-fold (*VvNF-YA6*). Nevertheless, some of them, i.e. *VvNF-YA7*, *VvNF-YB14*, *VvNF-YB15* and *VvNF-YC6*, exhibited decreased transcript levels (*P* < 0.05) at high concentration (1.0 and 2.0 %) of fructose (Fig. 8). These data showed that a reasonable number of *VvNF-Ys* displayed sugar-responsive expression pattern and their expression may be affected by the kind and concentration of exogenous sugars. The two *VvNF-Ys* (*VvNF-YA1* and *VvNF-YB7*) induced by sucrose could be involved in the biosynthesis and/or transport of sucrose in grape. The expression of *VvNF-YA1* and *VvNF-YB7* was promoted after exposure to fructose and glucose, respectively, indicating their potential roles in accumulation of these two soluble sugars during grape ripening.

## Discussion

NF-Y proteins have been revealed to be key factors in multiple physiological processes in plants [12, 13, 24, 25, 41, 42]. However, the function of most NF-Y proteins in grape are still unknown. Here we tried to take advantage of available data to explore and analyze the grape *NF-Y* family. Based on the results obtained from searching the Uniprot database by using PFAM and KOG IDs of conserved domains, we identified 32 previously predicted NF-Y proteins in PlantTFDB and two more members (Table 1). The two proteins, VvNF-YA1 and VvNF-YB13, consisted of more than 1000 amino acids, and their corresponding genes contained 13 and 11 exons, respectively (Additional file 2: Figure S2). Sequence analysis in InterPro revealed that VvNF-YA1 contained multiple functional domains, and the CBF signature (CBFB/NFYA, PFAM02045) was located between glutamic (E_1510_) and phenylalanine (F_1572_) next to the N-terminus. VvNF-YB13 contained only two domains, and the CBF domain (CBFD_NFYB_HMF, PF00808) was located between arginine (R_914_) and methionine (M_979_) close to the N-terminus as well. The phylogenetic analysis showed that VvNF-YA1 was homologous to VvNF-YA6, AtNF-YA4 and AtNF-YA7, while VvNF-YB13 was homologous to VvNF-YB14, AtNF-YB2 and AtNF-YB3 (Fig. 1). Altogether, VvNF-YA1 and VvNF-YB13 were regarded as the members of grape NF-Y family.

Evolutionary analysis could be used to predict potential functions of unknown members based on the known functions of those well-studied members of the same clade [3, 43, 44]. Therefore, an un-rooted phylogenetic tree based on sequences of NF-Y proteins from grape and *Arabidopsis* were constructed to explore the functions of VvNF-Ys (Fig. 1). For example, *AtNF-YB2* and *AtNF-YB3*, the homologous genes of *VvNF-YB13* and *VvNF-YB14* in *Arabidopsis*, were reported to promote flowering in response to inductive long-day condition [45], so the two *VvNF-YBs* might play roles in regulation of flowering in grape. More importantly, the genes required for flowering time control were generally expressed in leaf vascular tissue [45, 46], and our data showed that *VvNF-YB14* was differentially expressed in leaves (Fig. 6a). This result indicates that *VvNF-YB14* should be the candidate gene of particular interest for further study of flowering time control in grape.

Alterations in exon-intron structure or conserved domains would change the function of the gene or protein [1, 47]. Analysis of exon-intron structures revealed that most of *VvNF-YAs* had similar exon-intron organization pattern whereas *VvNF-YBs* and *VvNF-YCs* exhibited more variable and complicated structures (Additional file 2: Figure S2). Multiple alignments of NF-Y protein sequences among different species revealed the conservation of functional domains in VvNF-YAs (Fig. 2a). VvNF-YBs and VvNF-YCs, however, exhibit alterations in their functional domains, yet most of functionality required residues are conserved (Fig. 2b, c). The conservation of functional residues indicates the conserved functions of VvNF-Ys as their orthologs function in other plant lineages [47–52], whereas changes in protein sequences may imply the alterations of function. A good example is that *VvL1L*, the homolog of *AtL1L* in grape, has been reported to play a role in grape somatic embryogenesis [24, 25].

To further investigate the function of Vv*NF-Ys*, the expression patterns of *VvNF-Ys* in different grape organs and in response to various stresses were examined. Some of *VvNF-Ys* were found to respond to at least one kind of stress treatments. For instance, *VvNF-YA3* and *VvNF-YB18* were apparently induced by salt and PEG treatments (Fig. 3a and Table 2). *VvNF-YA3* was homologous to *AtNF-YA1* (Fig. 1), which was previously reported to function in seed development and could be induced by drought treatment [2, 49, 53]. Given that *VvNF-YA3* was mainly expressed in seed (Fig. 6c), it can help improve the resistance of grape seed to water deficiency. In addition, *VvNF-YA3* might be also involved in maturation of seed and dehydration signaling [53]. Intriguingly, *VvNF-YB18* was also revealed to respond to heat, PI and ABA treatments (Table 2). The results showed that some *VvNF-Ys* might be regulators of multiple biological processes. A certain number of *VvNF-Ys* were revealed to be involved in response to certain stress treatment. The expression of *VvNF-YB9* was down-regulated after the heat treatment but increased again during the following recovery process (Fig. 3a and Table 2). Interestingly, *VvNF-YB9* was predominantly expressed (>1.5-fold) in pulp and skin (Fig. 6c), suggesting a role in grape berries in response to environmental temperature stress. *VvNF-YB13* and *VvNF-YB17* only responded to PI and their expression seems to be genotype-dependent (Fig. 4 and Table 2). Phytohormone treatments showed that *VvNF-YA8* could respond to various hormones with increased transcript level in response to MJ and CD, and decreased transcript level to ABA (Fig. 5 and Table 2). The results indicated that *VvNF-YA8* might play different roles in different hormone signaling pathways. Emerging evidence suggests that hormone signaling pathways regulated by ABA and MJ play significant roles in the crosstalk between abiotic and biotic stress signaling [54]. *VvNF-YA8*, along with *VvNF-YB18* discussed above, could be proposed as promising candidate that involved in crosstalk between hormone and stress signaling pathways.

**Table 2:** Expression patterns of *VvNF-Ys* in response to specific stresses

Gene Name	Stress response	Expression pattern
*NF-YA1*	Sucrose, fructose	Up-regulated
*NF-YA3*	Salt, PEG	Up-regulated
*NF-YA6*	Fructose (2.0 %)	Up-regulated
*NF-YA7*	MJ, CD	Down-regulated
	Glucose, sucrose	Down-regulated
	Fructose (0.5 %)	Up-regulated
*NF-YA8*	MJ, CD	Up-regulated
	ABA	Down-regulated
*NF-YB9*	Heat stress	Down-regulated
	Recovery process after heat stress	Up-regulated
*NF-YB13*	*Plasmopara viticola* infection (with plant bearing *Rpv2*)	Down-regulated
*NF-YB17*	*Plasmopara viticola* infection (with plant bearing *Rpv2*)	Down-regulated
*NF-YB18*	Salt, PEG, cold (5 °C)	Down-regulated
	Heat stress	Up-regulated
	*Plasmopara viticola* infection (with plant bearing *Rpv1* or *Rpv2*)	Up-regulated
	ABA	Down-regulated
*NF-YC2*	Fructose (2.0 %)	Up-regulated

A common response of plants to abiotic stresses such as drought and salinity is the accumulation of sugars and other compatible solutes [55, 56]. The study of relationship between expression levels of *VvNF-Ys* and sugar contents showed that the changes of fructose contents and expression levels of the tested *VvNF-Ys* were highly consistent in at least four individual grape varieties (Fig. 7). Exogenous sugar treatments showed that expression of most *VvNF-Ys* were down-regulated in response to exogenous glucose and sucrose treatments (Fig. 8), except that *VvNF-YB7* was up-regulated by glucose (the peak was around 2.0-fold, *p* < 0.01) and sucrose (the peak was over 3.0-fold, *p* < 0.01). This suggested that *VvNF-YB7* could be involved in carbohydrate anabolism in grape. Notably, almost all the *VvNF-Ys* responded strongly to exogenous fructose treatment at the concentration of 0.5 % (Fig. 8). Among these genes, *VvNF-YC2* and *VvNF-YA6* responded to a higher concentration (2.0 %) of fructose likewise (Table 2), implying that they might play roles in fructose accumulation in grape berries ripening. Nevertheless, the expression of *VvNF-YA6* was suppressed by exogenous glucose and sucrose. All the results revealed that the regulatory networks of sugar accumulation or metabolism in grape are complicated, and the involvement of *VvNF-Ys* in grape berry sugar signaling still need more experimental evidence.

## Conclusions

In the present study, 34 *VvNF-Ys* were identified, and evolutionary, structural and expression analyses were conducted to reveal their possible biological roles in stress responses, development, and sugar metabolism. Comparison of NF-Ys from grape and *Arabidopsis* provided rudimentary insight on the function of less-studied *VvNF-Ys* in relation to their well-understood homologs. Furthermore, investigation of expression profiles showed that *VvNF-Ys* responded to various abiotic and biotic stresses as well as hormone treatments. Moreover, analysis of *VvNF-Ys* expression during grape berry development revealed that *VvNF-Ys* might play roles in fruit set, ripening and sugar accumulation in grape berry. Based on prediction and experimental data, the *VvNF-Ys* might be involved in responses to salt, drought, cold and pathogens and may also play significant roles in grape berry development as well as sugar accumulation. More significantly, *VvNF-Ys* probably function as regulators to mediate cross-talk between different signaling pathways. All these results may contribute to further functional investigation of grape *NF-Y* family.

## Methods

### Identification of *VvNF-Y* genes

The sequences of grape NF-Y proteins were obtained from the UniProt (http://www.uniprot.org/), using PFAM ID PF02045, PF00808 and KOG ID KOG0869, KOG0871, KOG1561 for NF-YA (HAP2), NF-YB (HAP3) and NF-YC (HAP5), as queries [2]. The obtained sequences were then compared with those from the PlantTFDB database v3.0 (http://planttfdb.cbi.pku.edu.cn/) [57]. All putative NF-Y proteins were further verified with the tool of InterProScan (http://www.ebi.ac.uk/Tools/pfa/iprscan/) to confirm the existence of the core domains. The corresponding sequences of *NF-Y* genes were acquired from the Grape Genome Browser (12×) (http://www.genoscope.cns.fr/externe/GenomeBrowser/Vitis/). The incomplete and redundant sequences were omitted.

### Structure and chromosomal localization

The locations of *NF-Y* genes on grape chromosomes were obtained from the Grape Genome Browser (12×) (http://www.genoscope.cns.fr/externe/GenomeBrowser/Vitis/). Gene structures (exon-intron structures) were visualized by alignment of cDNA sequences with corresponding genomic DNA sequences with the online tool of GSDS 2.0 (http://gsds.cbi.pku.edu.cn/).

### Alignments and phylogenetic analysis of *NF-Y* genes

Multiple sequence alignments of identified grape and *Arabidopsis* NF-Y proteins were conducted using ClustalX 2.0 [58]. Then the results were used to construct Neighbor-Joining tree using MEGA5.0 with the number of bootstrap replications being set at 1000 [59]. The sequences of *Arabidopsis* NF-Y proteins used for analyses were obtained from the PlantTFDB database v3.0 (http://planttfdb.cbi.pku.edu.cn/) and the *Arabidopsis* Information Resource (TAIR, https://www.arabidopsis.org/).

### Plant materials and sugar treatment

Grape (*V. vinifera*) seedlings were grown in the *Vitis* germplasm resources garden of Institute of Botany, the Chinese Academy of Sciences, Beijing, under natural conditions. For qPCR analysis of *NF-Ys* expression, grape berries and leaves at fruit set, veraison and fully ripe stages were sampled and ground into powder in liquid nitrogen before RNA extraction.

Grape suspension cells derived from embryogenic callus that was induced from whole flowers of ‘Chardonnay’ grape were used for exogenous sugar treatment. The suspension cells were cultured in 250 mL flasks filled with 50 mL of liquid CSM medium (MS basal medium supplemented with 0.5 g/L glutamic acid, 1 mg/L NOA, 5.0 mL/L glycerol and 20 g/L maltose), and shaken at 117 rpm at 27 ± 1 °C in the dark. All the suspension cells were sub-cultured every 7 days. For exogenous sugar treatment, glucose, sucrose and fructose were added to the media for final concentrations of 0.5, 1, and 2 % (w/v), respectively, at the time of 5 ~ 6 days (logarithmic growth phase of cells) after subculture. Then, the suspension cultures were centrifuged at 5000 rpm for 5 min, and the liquid media was removed. The collected cells were washed 3 ~ 4 times with sterile deionized water and subsequently ground in liquid nitrogen for RNA extraction. Each treatment replicated three times.

### RNA isolation and quantitative real-time PCR

Total RNA was extracted using the RNAprep Pure Plant Kit (TianGen, Beijing, China) according to the manufacturer’s instructions. RNA integrity was confirmed by electrophoresis on 1 % agarose gels, and concentration as well as quality of RNA were detected by NanoDrop 2000 Spectrophotometer (Thermo Scientific, MA, USA). The removal of genomic DNA and synthesis of the first strand cDNA was performed using HiScript Q RT SuperMix for qPCR (+ gDNA wiper) Kit (Vazyme, Nanjing, China). Quantitative real-time PCR (qPCR) was carried out using AceQ qPCR SYBR Green Master Mix (Vazyme, Nanjing, China) with the CFX96 System (Bio-Rad, CA, USA). The qPCR reactions consisted of a hold at 95 °C for 5 min, followed by 40 cycles at 95 °C for 10 s and 60 °C for 30 s. Melting curve was included to verify the specificity of each primer pair. Grape *Actin1* (accession no. AY680701) and *UBC* (accession no. EC922622) [60] were used as internal controls. The results were evaluated by the method of the 2^-ΔΔCt^ [61]. The data were obtained from three technological and biological replicates and are shown as mean values ± SD. The significance of differential expression between controls and treatments was examined by Student’s *t*-test with *P* < 0.05 and *P* < 0.01 indicating statistically significant and highly significant, respectively. Primers used for qPCR are listed in Additional file 5: Table S2.

### Microarray and transcriptome data analysis

Two databases, the ViTis Co-expression DataBase (VTCdb, http://vtcdb.adelaide.edu.au/Home.aspx) [62] and the Plant Expression Database (PLEXdb, http://www.plexdb.org/index.php), were searched for the probe sets of grape *NF-Y* genes. Finally, 16 probe sets, which were designed for Affymetrix GeneChip 16 K *Vitis vinifera* (Grape) Genome Array and Affymetrix GrapeGen *Vitis vinifera* Array, were found in both two databases and successfully matched to the 16 sequences of the 34 identified *NF-Y* genes (Additional file 3: Table S1).

Microarray data of grape *NF-Y* genes were obtained from PLEXdb (http://www.plexdb.org/index.php). Probe IDs of *NF-Y* genes were used as query items to search the Affymetrix GeneChip platform. The expression data for selected *NF-Y* genes were obtained and shown as heatmaps with a color scale indicating log2 expression values.

### Sugar extraction and HPLC analysis

Grape berries were collected and ground into powder in liquid nitrogen. The extraction of soluble sugars was conducted as previously described [63]. Around 100 mg of powder was fitted with 10 mL of methanol:chloroform:water (12/5/3; v/v/v), then sonicated for 30 s, and subsequently centrifuged at 1200 × g for 10 min. The supernatants were collected and diluted with water (5/3; v/v). Finally, 2 mL of the aqueous phase was evaporated and then dissolved in 0.8 mL of deionized water. The prepared samples were analyzed by HPLC with water as eluent (0.6 mL min^−1^).

## Abbreviations

ABA, abscisic acid; CBF, CCAAT binding factor; CD, cyclodextrin elicitor; Dav, day after veraison; HAP, heme activator protein; MJ, methyl jasmonate; NF-Y, nuclear factor Y; PI, *Plasmopara viticola* inoculation; PM, powdery mildew; qPCR, quantitative real-time polymerase chain reaction; TF, transcription factor

## Additional files

Additional file 1: Figure S1. Distribution of *NF-Y* genes on grape chromosomes. The *NF-Y* genes are ordered arranged according to their relative locations on chromosomes. (PDF 246 kb) Additional file 2: Figure S2. Exon-intron structures of grape *NF-Y* genes. Rectangles indicate exons, and broken lines represent introns. (PDF 219 kb) Additional file 3: Table S1. Probe sets from Affymetrix Microarray Platform for grape *NF-Y* genes used in microarray analysis following abiotic, biotic, and phytohormone treatments, and expression pattern assays in grape berry tissues. (PDF 100 kb) Additional file 4: Figure S3. Expression levels of *VvNF-Ys* and total sugar content in different grape varieties. *VvNF-YAs* (a), *VvNF-YBs* (b) and *VvNF-YCs* (c) are divided into three groups. Soluble sugars were extracted from five grape varieties (S1–S5) and then analyzed by HPLC with water as eluent. The total content was relatively stable in three successive years (unpublished data), so values for 1 year are given as reference. Black broken lines denote transcript levels of *NF-Y* genes, and blue broken lines indicate total sugar content in grape berries. (PDF 224 kb) Additional file 5: Table S2. Primers for quantitative real-time PCR analysis of grape *NF-Y* genes expressions in this study. (PDF 96 kb)
