# Supplementary material for: Genome-wide identification and characterization of the NF-Y gene family in grape (vitis vinifera L.)
Source: BMC Genomics. 2016 Aug 11;17:605. doi: 10.1186/s12864-016-2989-3 (PMC4982312; doi:10.1186/s12864-016-2989-3)
Supplement: Additional file 1: Figure S1. — Distribution of NF-Y genes on grape chromosomes. The NF-Y genes are ordered arranged according to their relative locations on chromosomes. (PDF 246 kb) [file 12864_2016_2989_MOESM1_ESM.pdf]

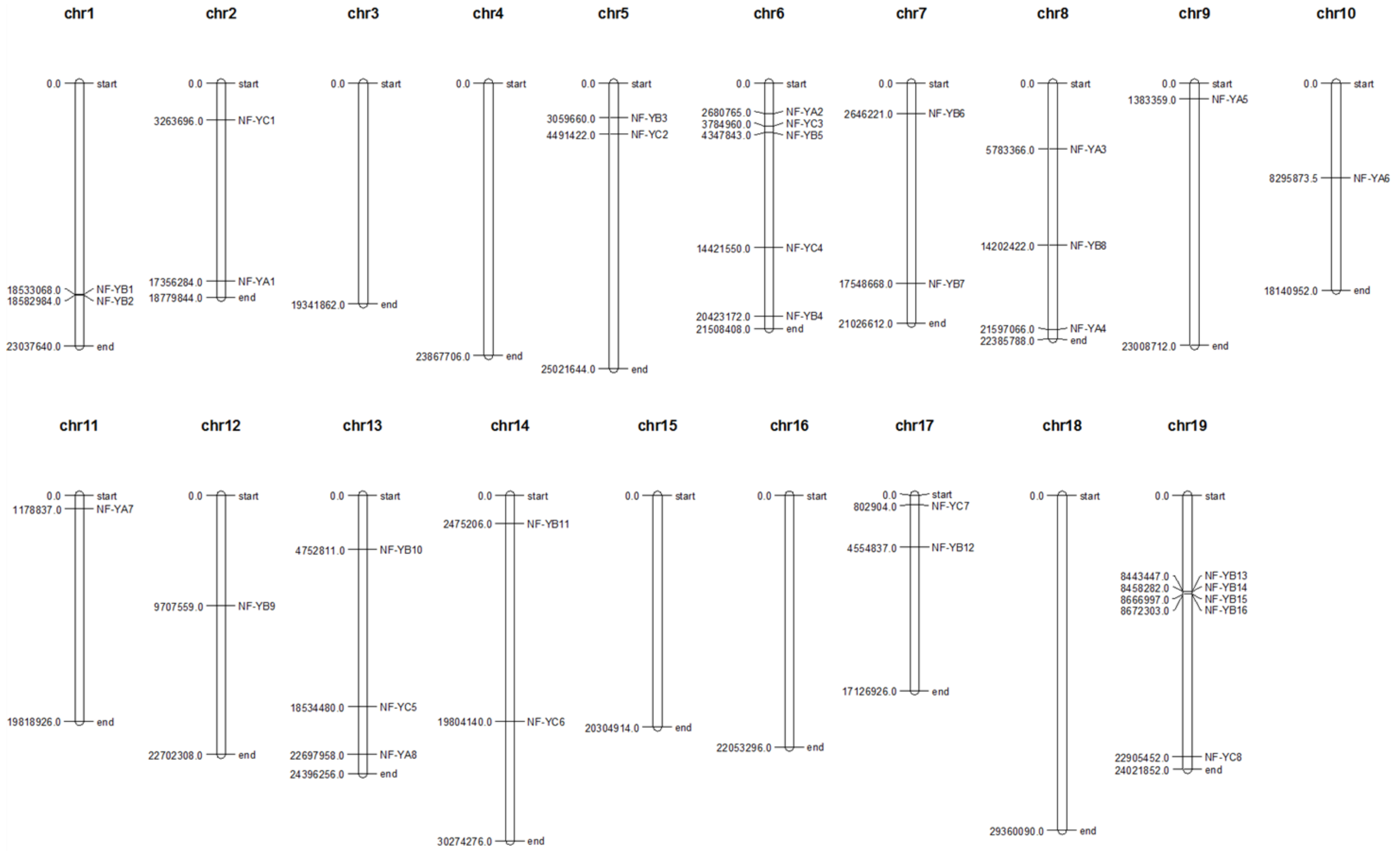
chr1
0.0 start
18533068.0 NF-YB1
18582984.0 NF-YB2
23037640.0 end
chr2
0.0 start
3263696.0 NF-YC1
17356284.0 NF-YA1
18779844.0 end
chr3
0.0 start
19341862.0 end
chr4
0.0 start
23867706.0 end
chr5
0.0 start
3059660.0 NF-YB3
4491422.0 NF-YC2
25021644.0 end
chr6
0.0 start
2680765.0 NF-YA2
3784960.0 NF-YC3
4347843.0 NF-YB5
14421550.0 NF-YC4
20423172.0 NF-YB4
21508408.0 end
chr7
0.0 start
2646221.0 NF-YB6
17548668.0 NF-YB7
21026612.0 end
chr8
0.0 start
5783366.0 NF-YA3
14202422.0 NF-YB8
21597066.0 NF-YA4
22385788.0 end
chr9
0.0 start
1383359.0 NF-YA5
23008712.0 end
chr10
0.0 start
8295873.5 NF-YA6
18140952.0 end
chr11
0.0 start
1178837.0 NF-YA7
19818926.0 end
chr12
0.0 start
9707559.0 NF-YB9
22702308.0 end
chr13
0.0 start
4752811.0 NF-YB10
18534480.0 NF-YC5
22697958.0 NF-YA8
24396256.0 end
chr14
0.0 start
2475206.0 NF-YB11
19804140.0 NF-YC6
30274276.0 end
chr15
0.0 start
20304914.0 end
chr16
0.0 start
22053296.0 end
chr17
0.0 start
802904.0 NF-YC7
4554837.0 NF-YB12
17126926.0 end
chr18
0.0 start
29360090.0 end
chr19
0.0 start
8443447.0 NF-YB13
8458282.0 NF-YB14
8666997.0 NF-YB15
8672303.0 NF-YB16
22905452.0 NF-YC8
24021852.0 end
