# Supplementary figures and images for: Genome-wide identification and characterization of the NF-Y gene family in grape (vitis vinifera L.)
Source: BMC Genomics. 2016 Aug 11;17:605. doi: 10.1186/s12864-016-2989-3 (PMC4982312; doi:10.1186/s12864-016-2989-3)

**NF-YA Subunits**

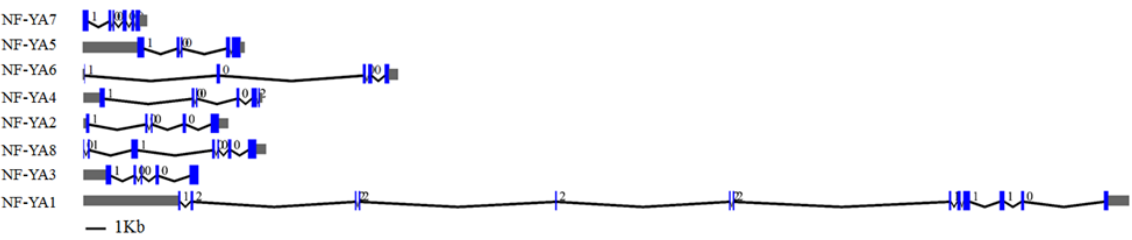

**NF-YB Subunits**

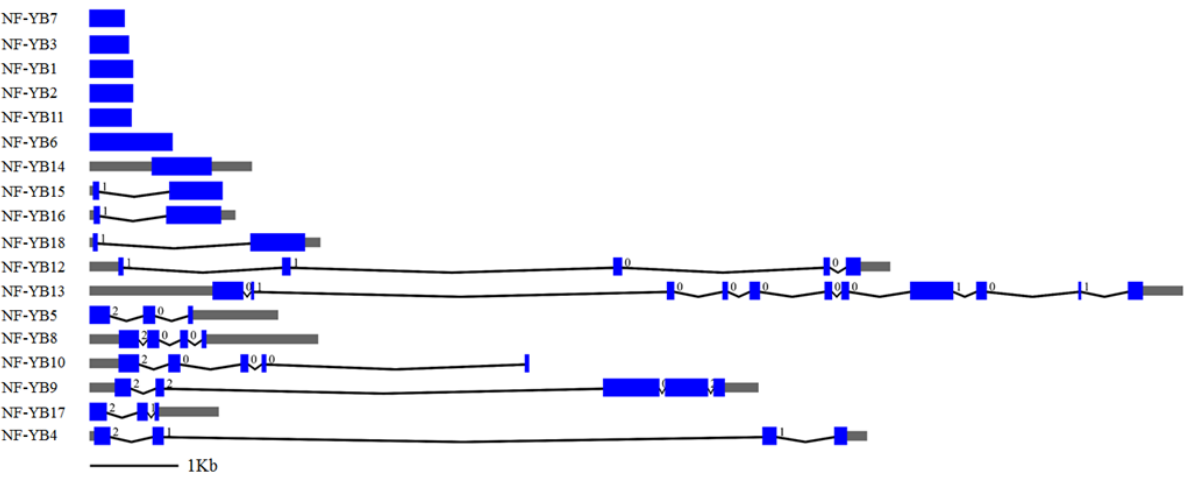

**NF-YC Subunits**

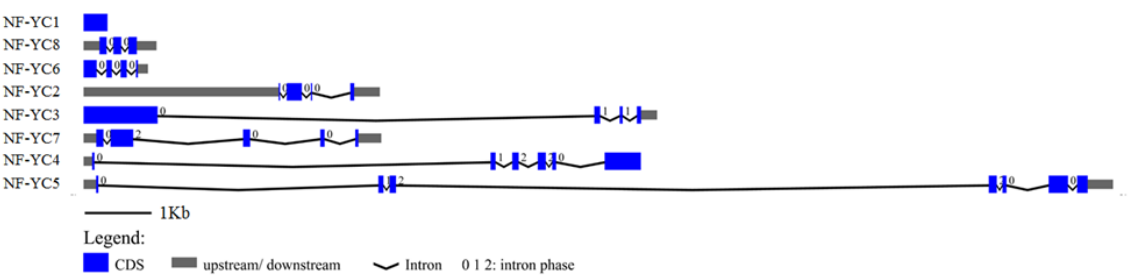

Supplement: Additional file 2: Figure S2. — Exon-intron structures of grape NF-Y genes. Rectangles indicate exons, and broken lines represent introns. (PDF 219 kb) [file 12864_2016_2989_MOESM2_ESM.pdf]

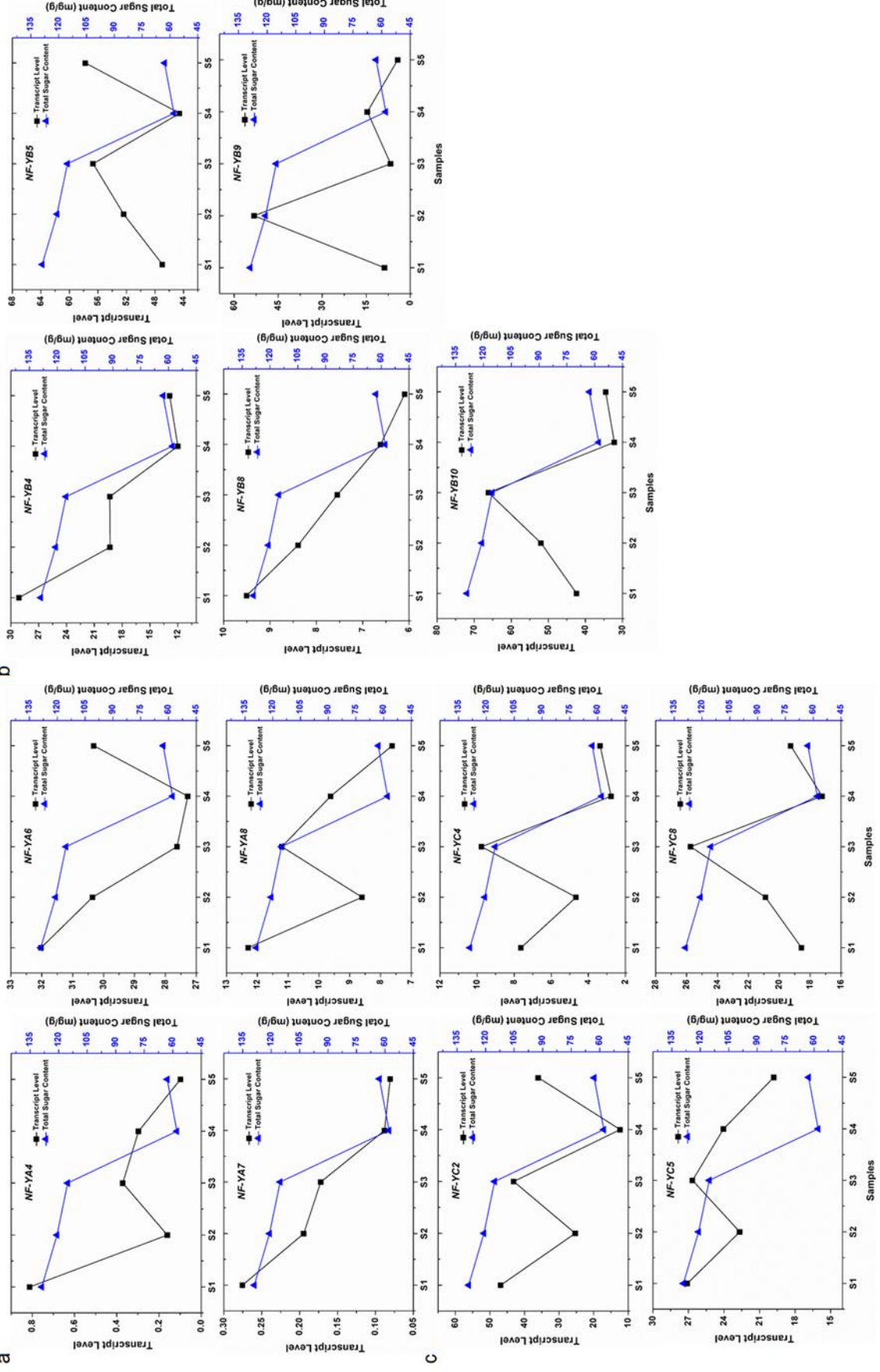

Supplement: Additional file 4: Figure S3. — Expression levels of VvNF-Ys and total sugar content in different grape varieties. VvNF-YAs (a), VvNF-YBs (b) and VvNF-YCs (c) are divided into three groups. Soluble sugars were extracted from five grape varieties (S1–S5) and then analyzed by HPLC with water as eluent. The total content was relatively stable in three successive years (unpublished data), so values for 1 year are given as reference. Black broken lines denote transcript levels of NF-Y genes, and blue broken lines indicate total sugar content in grape berries. (PDF 224 kb) [file 12864_2016_2989_MOESM4_ESM.pdf]
