# Supplementary material for: Genome-wide identification and characterization of the NF-Y gene family in grape (vitis vinifera L.)
Source: BMC Genomics. 2016 Aug 11;17:605. doi: 10.1186/s12864-016-2989-3 (PMC4982312; doi:10.1186/s12864-016-2989-3)
Supplement: Additional file 3: Table S1. — Probe sets from Affymetrix Microarray Platform for grape NF-Y genes used in microarray analysis following abiotic, biotic, and phytohormone treatments, and expression pattern assays in grape berry tissues. (PDF 100 kb) [file 12864_2016_2989_MOESM3_ESM.pdf]

Gene Name	Probesets ID		Identity (%)
	Grape *Vitis vinifera* 16K	*Vitis vinifera* GrapeGen	
NF-YA2	1618496_at	VVTU7693_at	95.4
		VVTU32730_at	100.0
NF-YA3	1615298_s_at	VVTU15501_at	100.0
	1613198_at	VVTU10245_at	100.0
	1613964_at		98.7
	1613912_at		98.7
NF-YA5	1621922_at	VVTU16239_at	100
NF-YA6	1615871_at	VVTU2545_at	96.9
NF-YA7	1620399_at	VVTU5429_at	100
NF-YA8	1621265_at	VVTU3152_at	99.7
NF-YB4	1617831_at	VVTU21324_at	95.2
		VVTU4637_at	95.2
NF-YB9	1613827_s_at	VVTU26075_x_at	98.0
NF-YB10	1616087_at	VVTU15088_s_at	95.2
NF-YB12	1607202_s_at	VVTU2955_at	100.0
NF-YB13	1611997_at	VVTU7943_at	99.6
NF-YB17	1617362_at	VVTU9459_at	98.0
NF-YB18	1614136_at	VVTU2237_at	99.1
NF-YC2	1616105_at	VVTU17073_at	99.7
NF-YC5	1609311_at	VVTU7457_s_at	99.5
	1613105_at	VVTU25126_at	100.0
NF-YC8	1607559_at	VVTU6141_s_at	99.5
