# Supplementary material for: Genome-wide identification and characterization of the NF-Y gene family in grape (vitis vinifera L.)
Source: BMC Genomics. 2016 Aug 11;17:605. doi: 10.1186/s12864-016-2989-3 (PMC4982312; doi:10.1186/s12864-016-2989-3)
Supplement: Additional file 5: Table S2. — Primers for quantitative real-time PCR analysis of grape NF-Y genes expressions in this study. (PDF 96 kb) [file 12864_2016_2989_MOESM5_ESM.pdf]

Gene name	Primer sequence (forward/reverse)	Product size (bp)
Actin 1	CAGCAGATGTGGATCTCAAA	59
	CTGTGGACAATGGAAGGAC	
UBC	GAGGGTCGTCAGGATTTGGA	75
	GCCCTGCACTTACCATCTTTAAG	
NF-YA1	TTTCCACAGGGATTTTGCGAGATA	149
	CTCTGCTCTGCCCTCAACATAC	
NF-YA6	CACAACTTGGGGCAGGACAT	108
	GTTGTGCAGGATAGGGCTGT	
NF-YA7	AGCCCGAAAGCCATATCTGC	144
	CCAGTATCAGAACTATGACAGCCA	
NF-YB4	GATCCTGTTGCTCTAAATGCCA	166
	GGCGAGCTGCAAAACAGAAG	
NF-YB5	TGGCATATGCAACTGAAAGAGAAGC	125
	GGCATTGTCAACTTTGCTTCCAC	
NF-YB7	AGCAGATCCTGCCACCTAATG	142
	TCCCCATTGACAGTCTTGCG	
NF-YB8	ACATCAAGGTCCTTACACGCA	92
	TTTCTTGGAGCATGCAGTCA	
NF-YB10	CAGCGAGGCTAGTGATAAGTGTC	150
	CCCCTGGTATCACCCTCTCTATATC	
NF-YB14	GGTCTCGTCGGCGATATTCC	139
	ACGACGTAGAGTCTCGACCA	
NF-YB15	GAAAGTGAGGTGGGTTGTGC	115
	CGCCAGGATTCAGAGCAGAG	
NF-YC2	CCAGGAATGAGCAGAAGCAAGA	134
	TGTGCAGCCCACATATTCCG	
NF-YC4	AGGAAGAAGCTCGACACACG	101
	ACTAGGAGAGGCACAGCCAA	
NF-YC5	AGATGAAGATCACCAGCACCAG	111
	TTCAGAAAGTGACCAGCCAGG	
NF-YC6	GAGCAGGATTTGTTGTGGCA	150
	TTCCTCGCTTGGGAGCATTT	
NF-YC8	GGTCTTGGGGCAGTTGTTGA	128
	GTATCTGTTTGCTGCTGTTGGG	
